# An Account or the Production of Epilepsy from Protracted Bathing in a Pond

**Published:** 1828-12

**Authors:** H. George Doyle

**Affiliations:** Mayslick, Ky.


ART. III.—*An account of the production of Epilepsy from protracted bathing in a pond.* By H. GEORGE DOYLE, M. D. of Mayslick, Ky.

On the 18th of August, 1827, I was called to visit the son of a gentleman of this county—a youth of about 14 years of age. On my arrival, I received the following history of his case:—About a month prior to the above date, he had gone to a neighbouring mill pond to bathe, in which he remained the principal part of the day, and returned at night, much exhausted, and enfeebled. The same night, an hour or two after he had retired, he sprang from his bed apparently much alarmed, screamed most violently, and for some minutes continued running and jumping about the room. After the paroxysm had subsided, his parents interrogated him on the subject of his fright,—to which he replied, that he had been pursued by Indians, who designed to destroy him. The father supposing that it was the effect of a dream, desired his son to compose himself, and return to bed. During this night he had two other paroxysms of the same description. The next night the family were again alarmed by a repetition of the attack, and from this time until I saw him, he suffered every night from this nocturnal visitation, until what to others is termed "nature's sweet restorer," was to him the bane of life.

The attacks at this time were sudden and without any premonition of their approach. Upon examination, I found his pulse full and tense, and his tongue considerably furred; he complained of pain in his head, and right side; his appetite was voracious; bowels rather disposed to constipation and the dejections indurated. I took 12 ounces of blood from his arm, which produced syncope, and ordered him 20 grains of calomel with 3 grains of emetic tartar, which operated freely upon the stomach and bowels, and affected the discharge of several very large lumbrici. His fits of screaming, &c. continued. On the morning of the 19th, gave him cal. ppt. 20 pulv. spigelia, grs. 10—and in the evening an infusion of senna and spigelia. The medicines operated well, producing a very copious discharges of bilious matter, and several worms. The pains in his head and side were considerably mitigated, his appetite improved, and his tongue got clean; but no effect was produced on his nocturnal fits of tossing and screaming. My attendance was now discontinued, and Dr. Bailey, who resided in the immediate neighbourhood, was employed. He introduced a seton near the occiput—and gave him active purges. For a few days, the disease appeared to yield—the number and violence of the paroxysms becoming less. The seton, however, soon lost its efficacy, and seemed rather to become an additional cause of irritation.

About this time we had a visitation from one of those charlatans, who, under the *imposing* name of Indian doctor, occasionally make their appearance like an epidemic. The unhappy sufferer was, by his despairing and too credulous father, consigned to the care of this monster, who dosed and drenched him with his Indian nostrums, until the "vital spark" was nearly extinguished.

He then became the patient of my friend Dr. Scudder, who visited him January 2d, 1828. His pulse was full and tense, and 80 in the minute,—appetite good—tongue clean—complained of no pain—bowels rather torpid. He took 12 ounces of blood from his arm—applied sinapisms to his feet—and cloths dipped in cold water to his head; but the paroxysms continued through the following night. 3d, He complained of nausea, and took an emetic. 4th, Took 20 grains calomel in the morning, and an infusion of senna and spigelia at night. From this time until the 1st of February, took active mercurial purges every night. They occasioned very copious bilious discharges, but made no impression upon the symptoms. On the 2d, lost 12 ounces of blood from a branch of the temporal artery, and took 70 drops tinct. opii. at night, about an hour before the expected paroxysm. On the following night the tinct. opii. was repeated, but it appeared to have an injurious effect. He was now placed upon an alterative course of mercury, which was continued for a fortnight, when it began to produce violent griping pains in the bowels. His gums remained unaffected. Opium was now combined with the calomel, but to no purpose; the painful affections of the bowels continued, and the screaming and jumping returned every night with unabated violence. The blue pill was then substituted for the calomel and opium, and continued until the last of March. The patient had now arrived at the very summit of human suffering; for, in addition to the increased violence of the nocturnal paroxysms, (which were from 7 to 10 in the course of the night,) they would frequently terminate in Epileptic convulsions, and his mind was fast falling into idiocy.

On the first of April, I again saw this patient, in company with Dr. Scudder, and spent an entire night with him, during which he had five attacks, the last of the Epileptic form. In this his whole body was most violently and horribly convulsed. I kept my fingers as well as I could, upon the radical artery during this attack, to ascertain, if possible the effect produced upon the circulation, and found his pulse not perceptible. In about two minutes after the spasmodic distortions of the body had subsided, he was perfectly breathless, and to all appearance dead. We were alarmed at his situation, and, in a moment of anxiety, I threw upon him a pitcher of cold water. It produced a most violent fit of screaming, from which he soon recovered, and was somewhat incensed, at the means employed for his rescussitation.

From the whole history and treatment of this case, I was induced to suggest to Dr. S. the propriety of trying the nitrate of silver, assisted by the sulphate of quina, and occasional mild evacuants, for I supposed the symptoms might depend on a morbid sensibility of the stomach and bowels. This treatment, beginning with the fourth of a grain, was continued until six grains of the nitrate of silver were given in the 24 hours, without the least sensible effect being produced upon the disease, when like any other prescription, it was abandoned in despair.

I saw nothing more of this patient from that time, until the first day of this month, (November 1828,) when I visited him again. His father informed me, that the symptoms had become more distressing, and that he now thought of no relief for him but in death,—that his intellectual powers were seriously injured,—that the frequency and violence of the paroxysms were increased by exercise,—that he generally had one or two attacks in the day, and that when awake, he was admonished of their approach by a considerable degree of vertigo. He complained of a dull, heavy pain in the head, his pulse continued full and somewhat tense, between 70 and 80,—his countenance wore a melancholy aspect, and his eye was rather heavy, though he was generally disposed to loquacity.

It would be interesting to ascertain what effect tying one of the carotid arteries would have in this case. Though I was induced for a length of time to hope, that the complaint was primarily an affection of the digestive organs, and would readily yield to a course of alterative treatment, I am now as firmly persuaded, that it is entirely dependant on cerebral irritation, and that probably nothing short of the operation of which I have spoken, will probably relieve him. Should he submit to the operation, I have much hope that the result will be such as to suggest the utility of tying those arteries, in other obstinate cases of Epilepsy.

*November*, 1828.

### REMARKS BY THE EDITOR.

The foregoing case presents us with a train of cerebral symptoms, eventuating in hopeless Epilepsy, obviously produced by remaining too long in the water in the summer time;—a practice to which the boys of this country are addicted: it seems worthy, therefore, of being recorded. While few diseases are more constant in their phenomena, scarcely any is more diversified in its remote causes. With respect to the proposition of Dr. Doyle, to tie one of the carotid arteries, I would say, that it might be well first to try the effect of compressing those vessels. Could the proposed operation have succeeded in the early stages of the diseases, it by no means follows, that it would afford the same result after sufficient time had elapsed for the production of organic læsions of the brain or its membranes. But it is far from being settled, whether in epilepsies attended with manifest determinations of blood to the brain, the sanguiferous irregularity should be regarded as the cause or the effect of the encephalic irritation, which occasions the convulsions. That turgescence of the vessels of the brain, is *preceded* by some morbid affection of that organ as its cause, is extremely probable; but the convulsions may be the *immediate* offspring of the turgescence, and in that case, if it should be prevented by pressure or obliteration of a part of the vessels leading to the head, the *spasms* might be cured; but it would not follow, that the patient would recover. The affection of the nervous matter, which was the primary link in the chain of morbid actions might, with a different train of symptoms, continue to a fatal termination.

